# The Impact of Bisphenol A on Fertility, Reproductive System, and Development: A Review of the Literature

**DOI:** 10.1155/2019/4068717

**Published:** 2019-04-10

**Authors:** Ewa Matuszczak, Marta Diana Komarowska, Wojciech Debek, Adam Hermanowicz

**Affiliations:** Pediatric Surgery Department, Medical University of Bialystok, Poland

## Abstract

Bisphenol A (BPA) has been used since the 1950s, in food packaging, industrial materials, dental sealants, and personal hygiene products. Everyone is exposed to BPA through skin, inhalation, and digestive system. BPA disrupts endocrine pathways, because it has weak estrogenic, antiandrogenic, and antithyroid activities. Despite the rapid metabolism, BPA can accumulate in different tissues. Many researchers proved the impact of BPA on human development, metabolism, and finally reproductive system. There is increasing evidence that BPA has impact on human fertility and is responsible for the reproductive pathologies, e.g., testicular dysgenesis syndrome, cryptorchidism, cancers, and decreased fertility in male and follicle loss in female.

## 1. Introduction

Bisphenol A (BPA) (2,2-bis(4-hydroxyphenyl)propane; BPA) is an organic synthetic compound, belonging to the group of diphenylmethane derivatives and bisphenols. It has been used since the 1950s, in food packaging, industrial materials, dental sealants, and personal hygiene products [1]. Everyone is exposed to BPA through skin, inhalation, and digestive system. It is poorly soluble in water and soluble in organic solvents. High temperatures cause the release of free BPA [2]. Epoxy resins containing BPA are used to line water pipes, as coatings on the inside of many food and beverage cans and in making thermal paper, such as that used in sales receipts. In 2015, an estimated 4 million tons of BPA chemical was produced for manufacturing polycarbonate plastic, making it one of the highest volumes of chemicals produced worldwide [3]. BPA absorbed by humans is metabolized by the liver, with half-life of 6 h, and excreted with the urine in 24 h [1, 2]. BPA disrupts endocrine pathways, because it has weak estrogenic (1000–100,000-fold less than that of estradiol), antiandrogenic, and antithyroid activities. Despite the rapid metabolism, BPA can accumulate in different tissues [3]. Even though the European Union and Canada have banned BPA use in baby bottles, it was found in the serum of babies fed by breast and fed by bottle alike. This finding shows our ubiquitous vulnerability to BPA [3]. Many researchers proved the impact of BPA on human development, metabolism, and finally reproductive system [4].

BPA has been tested as an artificial estrogen already in the early 1930s. Diethylstilbestrol (DES), a structurally similar compound, was used as a synthetic estrogen drug in women and animals until it was banned in the 1970s, due to its risk of causing cancer [1–5]. Typically phenol-containing molecules similar to BPA are known to exert weak estrogenic activities; thus it is also considered an endocrine disrupter (ED). BPA is a xenoestrogen, exhibiting estrogen-mimicking, hormone-like properties, because of the similarity of phenol groups on both BPA and estradiol, which enable this synthetic molecule to trigger estrogenic pathways in the body. There is increasing evidence that BPA has impact on human fertility and is responsible for the reproductive pathologies, e.g., testicular dysgenesis syndrome, cryptorchidism, cancers, and decreased fertility in male and follicle loss in female [4, 6–8]. BPA has been found to bind to estrogen receptors (ERs), ER*α*, and ER*β*, but still it is less potent than estradiol [4, 6–8]. BPA is not only a selective estrogen receptor modulator (SERM) but also a partial agonist of the ER [4, 6–8]. It can also bind to the androgen receptor (AR) and act as its antagonist when its level is high [4, 6–8]. Moreover BPA has been found to affect Leydig cell steroidogenesis, including affecting 17*α*-hydroxylase/17,20 lyase and aromatase expression and interfering with LH receptor-ligand binding [4, 6–8]. In 2017 the European Chemicals Agency has listed BPA as a substance of very high concern, due to its properties as an endocrine disruptor.

## 2. Material and Methods

Pubmed (http://www.ncbi.nlm.nih.gov/pubmed) searches for the years 2000–2018 were conducted using the following key words: BPA, bisphenol A, fertility, reproduction, female, and male. We focused on manuscripts published in 2000–2018, to expand upon previous review papers on the same topic [4, 5]. Additionally, references included in other review papers were examined for relevant information. 187 studies were identified, among them 97 were researching human populations.

## 3. Female

Infertility in women results from various factors, environmental, endocrine, lifestyle, and also physical problems, and nowadays can reach up to 30% worldwide [8–10]. Environmental factors cause the exposure to endocrine disrupting chemicals, which can mimic or block the endocrine activity of endogenous estrogen and affect reproduction [11]. BPA is a xenoestrogen, exhibiting estrogen-mimicking, hormone-like properties. Many animal studies show that BPA as a toxicant has adverse effects on fertility, delays the onset of female puberty, and has influence on estrous cycle [4]. BPA has been found to bind to both of the nuclear estrogen receptors (ERs), ER*α*, and ER*β*. BPA can both mimic the action of estrogen and antagonize estrogen, indicating that it is a selective estrogen receptor modulator (SERM) or partial agonist of the ER [4, 6–8]. BPA seems also to interfere with the steroidogenesis process by reducing StAR and P450 aromatase, whereby the production of E2 is blocked [12]. These changes are correlated with an increase in follicular atresia and seen in human diseases that lead to infertility, polycystic ovary syndrome, and endometriosis [12]. At the level of ovary “BPA interfere with histone modification, leading to the downward adjustment of lhcgr mRNA levels, and probably with global methylation because of its ability to interfere with the dnmt expression” [12].

Studies on animals showed that the negative impact of BPA on female fertility derives from impaired cytoskeletal dynamics of oocyte, induction of oxidative stress, increased DNA damage, and changing of the status of epigenetic modifications in oocyte [13].

### 3.1. Oocyte

The female germ cells produce a primordial germ cell, which then undergoes mitosis, forming oogonia. During oogenesis, the oogonia become primary oocytes. BPA affects the maturing of the oocyte [8, 12]. Infertile women have higher serum BPA levels compared to fertile women. In women who undergo in vitro procedure, a higher level of BPA in the urine negatively correlated with oocytes maturation and peak concentrations of estradiol [9]. Most probably, the every-day exposure to BPA diminishes the ovarian reserve [10]. Also in another cohort study by Ehrlich et al., higher urinary BPA concentration correlated with the poor oocyte yield and with lower serum E2 [14]. Bloom et al. also observed association between higher BPA and lower serum level of E2 but did not find the association between BPA and the number of oocytes retrieved per cycle [15]. According to Fujimoto et al., in women undergoing intracytoplasmic sperm injection, a higher concentration of BPA in the serum was connected with a decreased probability of mature oocytes [16]. Still Zhao et al. did not find correlation between BPA and E2 in healthy adult women [17]. But in women with polycystic ovary syndrome (PCOS) Kandaraki et al. found “a significant association between BPA and elevated androgen concentrations” [18]. Still, not all epidemiological studies report an association between BPA exposure and fertility outcomes. Buck Louis et al. did not find correlation between total urinary BPA concentrations and impaired fecundity in healthy women [19]. According to other human studies, preconception concentrations of BPA in female urine were associated with decreased fecundability [20]. Nevertheless, all these studies did not take into account potential modifying factors, such as coexposure to other chemicals.

### 3.2. Hypothalamic-Pituitary-Ovarian Axis

The uterus responds to the changing hormone levels produced by the brain as well as the ovaries. This process is initiated in the hypothalamus through the production and release of GnRH, which leads to FSH and LH release from the anterior pituitary gland. As a result, oocyte development takes place in the ovaries, and estradiol is produced from the growing ovarian follicle. BPA can affect the hypothalamic system. The hypothalamic-pituitary-ovarian axis controls the ability of the mammalian female to ovulate and to prepare the reproductive organs to support potential pregnancy. BPA exposure resulted in the decrease of the reproductive capacity and delay or elimination of puberty [1]. In rats, in utero BPA exposure resulted in irregular estrous cycles in mature animals [21–23]. The exposure to 500 *μ*g/kg/day BPA in rats leads to anovulation and infertility [22]. Moreover, BPA may target GnRH neurons and as a result cause the decrease in GnRH mRNA expression [23, 24].

Only small number of human studies concerning the association between BPA exposure and pituitary outcomes can be found. Miao et al. reported “a positive association between creatinine adjusted urine BPA levels and PRL and a negative association with FSH levels in women exposed to BPA in their work place” [25]. In contrast, Souter et al. found “no association between specific-gravity adjusted BPA levels and day-3 FSH levels in women undergoing IVF treatments” [26]. These dissimilar results may be due to the differences between two cohorts. The first examined female workers are from manufacturers of epoxy resin in China and workers from garment factories; the latter are women undergoing infertility treatments at the Fertility Center.

BPA interfere with the synthesis of gonadotropins. According to Brannick et al. mice after the administration of BPA had decreased levels of “gonadotropin mRNA, Gnrhr, and Nr5a1, key components of gonadotropin synthesis” essential for signal transduction between the pituitary and the hypothalamus [27].

### 3.3. Polycystic Ovary Syndrome

Nowadays the polycystic ovary syndrome (PCOS) is the most common women endocrinopathy. PCOS symptoms are hirsutism, dysfunction of menstrual cycle, and infertility caused by hyperandrogenism, anovulation, and insulin resistance [28]. Both human and animal studies have suggested a possible role of BPA in PCOS aetiopathogenesis [28–35]. Several studies show that women with PCOS had a higher BPA concentration, than healthy women [28–35]. Young Rodents exposed to BPA, when matured, developed PCOS-like syndrome [28]. Moreover a negative correlation between the urinary BPA concentration and the antral follicle count in women with PCOS was found [26]. Also BPA levels were negatively correlated with AMH and day-3 FSH levels, but the differences were statistically nonsignificant [8].

### 3.4. Oviduct and Uterus

The oviduct and uterus are part of the female reproductive system and are specialized organs that serve to transport oocytes to the site of fertilization and implantation, to support fetal growth and nourishment. BPA has also effects on oviduct and uterus, triggering the formation of progressive proliferative lesions [1]. The evidence on the effects of BPA exposure on the oviduct is based on experimental studies in mice. The exposure of prenatal mice to the low dose BPA resulted in formation of progressive proliferative lesions in the oviduct and remnants of the Wolffian duct in mature animals [36]. Other studies indicate that high dose BPA exposure during prenatal life delayed development and transport of the embryo compared to controls [37]. This data indicates that gestational BPA exposure affects morphology and function of oviduct. Both prepubertal and gestational mice treated with BPA had proliferative lesions in the oviduct, and prenatal exposure of rodents to BPA caused atypical hyperplasia and stromal polyps of the uterus and endometriosis [36–38]. In other experimental studies, exposure to BPA diminished the uterine receptivity, so important for implantation of an embryo [36, 39–45]. These findings were only partially confirmed in human so far [41]. According to Ehrlich et al., in women undergoing in vitro fertilization, higher urinary BPA was connected with higher implantation failure, but this trend turned out to be statistically nonsignificant [46]. Sugiura-Ogasawara et al. found higher level of serum BPA in women who experienced recurrent miscarriages, and furthermore “there was a trend of higher BPA in the women with abnormal embryos” [47]. According to one study by Cantonwine et al., there is also association between BPA level and premature delivery [48]. Contrary to this observations two experimental studies report no effect of low or high doses (122 mg/kg/day) of BPA, on the number of implantation sites [45, 46].

### 3.5. Gene Expression

BPA can also affect the gene expression [49]. According to Caserta et al. who investigated women affected by primary infertility “a positive correlation was found between BPA levels and ESR1, ESR2 (nuclear receptors), AR (androgen receptor), AhR (hydrocarbon receptor), and PXR expression (pregnane X receptor), while PPAR*γ* (peroxisome proliferator-activated receptor gamma) expression did not show any meaningful difference” [50]. These findings support the hypothesis that BPA acts on nuclear receptor, disturbing hormone response pathways and steroidogenesis, affecting female infertility [1].

### 3.6. Ovary

The ovary progresses through many stages beginning in the prenatal period through menopause; it secretes hormones that play a role in the menstrual cycle and fertility. Studies on animals found also the influence of in utero or neonatal BPA exposure on ovary, which has changed morphology and histology when compared to controls. Experimental studies have shown that BPA also affect the oocyte and granulosa cells, essential for oocyte survival and nourishment [1]. In rodents and lambs exposed to BPA postnatally, the ovarian follicular reserve was reduced, with “a decline in the stock of primordial follicles, increase in antral atretic follicles, higher incidence of multiple oocyte follicles, and lower ovarian weight” [24, 51]. In other studies “murine ovarian granulosa cells cultured with BPA had increased granulosa cell apoptosis, decreased granulosa cell viability, and increased follicular atresia” [52].

### 3.7. Progeny

Maternal or paternal exposure to BPA can affect the birth weight of newborns [14]. Miao et al. observed that babies born to mothers exposed to BPA had significantly lower birth weight. Lower birth weight was also observed in children of fathers exposed to BPA but in this case the difference was not statistically significant [53]. Moreover Chou et al. found that a higher level of BPA in mother serum increased a risk of having a male infant with low birth weight [54]. Contrary to that observation, Philippat el al. found “a positive association between maternal BPA and both weight/size and increased head circumference with higher maternal urinary BPA” [55]. Earlier studies from 2008 did not find correlation between birth weight of a child and maternal serum or urine BPA [56, 57].

## 4. Male

It is not debatable that BPA disrupts spermatogenesis [8, 58]. At high concentrations, BPA binds to and acts as an antagonist of the androgen receptor (AR) [59]. In addition to receptor binding, the compound has been found to affect Leydig cell steroidogenesis, including affecting 17*α*-hydroxylase/17,20 lyase and aromatase expression and interfering with LH receptor-ligand binding [59]. Among couples in need of treatment of infertility, in 98% of patients, BPA was found in the urine samples, and its level negatively correlated with sperm count and motility [60]. In vitro BPA has also been linked to reduction of sperm reserves, shorter transit time of sperm, and lower mitochondrial activity [61, 62]. The same effects of BPA were noticed in rats and mice in vivo [63, 64]. Also higher rate of apoptosis of Sertoli cells was recognized [65]. In addition BPA as a toxicant alters the energy metabolism [66]. BPA exposure is related to a decrease in the activity of the antioxidant system, resulting in oxidative stress, the most common cause of damage to the sperm [67, 68]. However, we should emphasize that BPA exposure is not the only disruptor of sperm production. Still, there are some observations yielding contrary results; e.g., a large study by Chen did not prove the association between BPA and infertility in men with idiopathic infertility [69]. This study subjects were volunteers consecutively recruited from affiliated hospitals. They were the male partners of couples with problems with conception. In another cross-sectional study of healthy men recruited for military service, urinary BPA concentrations were inversely associated with progressive sperm motility but, there were no associations of BPA with other sperm parameters [58]. Meeker et al. explored the association of urinary BPA concentrations with semen parameters and DNA damage in male partners of subfertile couples and reported that “urinary BPA concentrations were negatively associated with sperm concentration, normal morphology, and sperm DNA damage” [70].

### 4.1. Testosterone Levels

Testosterone plays a key role in the development of male reproductive tissues such as testes and prostate, as well as promoting secondary sexual characteristics. Nakamura et al. in their experiment on rats found that higher doses of BPA positively correlated with the decrease of serum levels of testosterone and LH generating hypogonadotropic hypogonadism [71]. Moreover the expression of GnRH receptor was higher, and according to Wisniewski et al. “the observed pattern of gene expression is indicative of an attempt by the pituitary to reestablish normal levels of LH, FSH, and testosterone serum concentrations” [6]. According to Meeker et al., males with elevated urinary BPA had higher FSH and lower inhibin B levels, and BPA exposure “was associated with a higher FSH:inhibin B ratio and a lower estradiol:testosterone ratio” [70]. Mendiola et al. found that increase in BPA urine level correlated also with decreased free androgen index, and this association was statistically significant [72]. Many authors stressed the association of exposure to phenols and idiopathic male infertility [69, 73].

### 4.2. Progeny

In prenatal life our drug metabolizing system is immature, and moreover the placenta is not a barrier to maternal BPA [74]. Higher levels of BPA prenatally are found to be connected with the lower birth weight and smaller size for gestational age (SGA), more expressed in males [54]. Contrary to that, Lee et al. observed association between high birth weight and elevated levels of maternal BPA [75]. In a study by Bloom a higher level of paternal but not maternal serum BPA was connected with reduced embryo quality, which suggests “a role for sperm quality related to BPA exposure of the father on early reproductive development in the offspring” [76].

### 4.3. Sexual Function

Sexual function, erectile function and ejaculation in male, is regulated by complex mechanisms. Several central and peripheral neurological factors in addition to molecular, vascular, psychological, and endocrine factors are involved. It is worth noting that BPA exposure probably can also influence sexual function of men. In China workers exposed to BPA, self-reported sexual function was significantly decreased when compared with the control group [77, 78].

### 4.4. Cryptorchidism

Undescended testicle, with an incidence of 2 to 5% in boys born on term, is one of the most common genitourinary tract malformations in males, leading to surgical intervention, which does not completely do away with higher risk of infertility and malignancy. During the process of descending to the scrotum, which is starting in prenatal life, testicles are guided by the gubernaculum, and this process should be finished in boys born on term. Some authors link the BPA with cryptorchidism. The aetiology of cryptorchidism is multifactorial, genetic, hormonal, and environmental factors playing a role [79–82]. According to Komarowska et al., higher levels of serum BPA in boys with unilateral cryptorchidism “reflect the continuous exposure to BPA in our patients, connected with environmental sources” [3]. Contrary to that, other authors found that levels of uBPA are similar in boys with cryptorchidism and with testicles in the scrotum. Virtanen et al. did not find correlation between bisphenols in the placenta and interrupted testicular descent [83]. Moreover Hosie et al. studying serum levels of BPA did not find statistical differences between boys with cryptorchidism and controls [84]. A study by Fenichel et al., investigating cord blood in newborn males with or without their testicles in the scrotum, found a positive correlation between unconjugated cord blood BPA and total T and inhibin in controls [85].

In boys with cryptorchidism, according to Chung, “the reduction in germ cell count starts as early as 6 months of age and is dependent on the position of the testis” [86]. Study by Wilkerson et al. who compared fertility potential of undescended testes by age groups in children revealed that “the higher the testicular position at the time of treatment, the fewer the number of germ cells” [87]. According to human studies “spermatogenic index decreases significantly by 9 months of age”' so operation of undescended testes at this age or before may stop testicular degeneration and improve chances for future fertility [83]. Moreover, Tasian et al. reported “a significant 2% risk per month of severe germ cell loss and 1% risk per month Leydig cell depletion for each month a testis remains undescended” [88]. According to the same research “the odds of germ cell loss almost double for each age range at the time of orchidopexy” [88].

### 4.5. Genital Abnormalities

The human fetus is undifferentiated sexually until 8^th^ week of gestation and contains both male and female genital ducts. In male Wolffian structures differentiate into the vas deferens, epididymis, and seminal vesicles, the genital tubercle enlarges to form the penis, the genital folds become the shaft of the penis, and the labioscrotal folds fuse to form the scrotum. Differentiation occurs during 12-16 week of gestation and is regulated by testicular hormones. Because BPA is an antiandrogenic disruptor, more genital abnormalities could be expected in boys born to parents exposed to BPA. According to Miao et al. “boys from BPA exposed parents had shorter AGDs (anogenital distance), and boys from exposed mothers had a statistically significant correlation to BPA exposure, in both pre- and postpubertal analyses” [53].

### 4.6. Male Fertility

Sperm production in the testis depends on equilibrium between division and loss of germ cells. During spermatogenesis, apoptosis limits the quantity of germ cells and eliminates those which carry DNA mutations, occurring through chromosomal crossing over during the first meiotic division. Cohort studies investigating the correlation between male urinary BPA concentrations and couple reproductive outcomes often yield opposing results. Dodge et al. examined “the associations of paternal urinary BPA concentrations with fertilization, embryo quality, implantation, and live birth” among couples who underwent intrauterine inseminations and in vitro fertilization cycles and found no correlation [89]. Buck Louis et al. did not find association between paternal urinary BPA concentrations and time to pregnancy [19]. However, in the last study, higher paternal urinary BPA concentrations were significantly associated with fewer male births. Contrary to that, according to Radwan et al. higher urinary concentration of BPA increases the percentage of immature sperm [90]. This study provides evidence that exposure to BPA is associated with poorer semen quality [90]. There is more reliable evidence in relation to the negative effect of BPA on sperm quality and motility [91], but according to the same review of in vitro, in vivo, and epidemiological studies, “no unambiguous results were obtained in relation to the evaluation of associations between BPA and implantation failure in women, (…) sexual dysfunction in men, impact (…) on birth rate, birth weight and length of gestation” [91].

The effects of BPA exposure on fertility, reported by different authors, are dependent on the study design, timing and route of exposure to BPA, species being examined, and dose of BPA. Other modifying factors, such as study location and study sample may play a role. Studies on human population have certain limitations; e.g., different populations have various exposure levels of BPA, so comparing the results from the literature, one should know all confounding factors, since it is well known that the health outcomes are affected by many determinants. Still the literature on this topic is growing, so does our knowledge.

## 5. Conclusion

Broadening our knowledge of the effects of BPA may urge the reduction of its use and so lessen its impact on human fertility, reproductive system, and development.

## References

[1] Huo X., Chen D., He Y., Zhu W., Zhou W., Zhang J. (2015). Bisphenol-a and female infertility: a possible role of gene-environment interactions. *International Journal of Environmental Research and Public Health*.

[2] Vandenberg L. N., Chahoud I., Heindel J. J., Padmanabhan V., Paumgartten F. J. R., Schoenfelder G. (2012). Urinary, circulating, and tissue biomonitoring studies indicate widespread exposure to bisphenol a. *Ciencia & Saúde Coletiva*.

[3] Komarowska M. D., Hermanowicz A., Czyzewska U. (2015). Serum bisphenol a level in boys with cryptorchidism: a step to male infertility?. *International Journal of Endocrinology*.

[4] Rochester J. R. (2013). Bisphenol A and human health: a review of the literature. *Reproductive Toxicology*.

[5] Gregoraszczuk E. L., Ptak A. (2013). Endocrine-disrupting chemicals: some actions of pops on female reproduction. *International Journal of Endocrinology*.

[6] Wisniewski P., Romano R. M., Kizys M. M. L. (2015). Adult exposure to bisphenol A (BPA) in Wistar rats reduces sperm quality with disruption of the hypothalamic-pituitary-testicular axis. *Toxicology*.

[7] Lucas B., Fields C., Hofmann M.-C. (2009). Signaling pathways in spermatogonial stem cells and their disruption by toxicants. *Birth Defects Research Part C - Embryo Today: Reviews*.

[8] Zhou W., Fang F., Zhu W., Chen Z., Du Y., Zhang J. (2016). Bisphenol A and ovarian reserve among infertile women with polycystic ovarian syndrome. *International Journal of Environmental Research and Public Health*.

[9] Mok-Lin E., Ehrlich S., Williams P. (2010). Urinary bisphenol A concentrations and ovarian response among women undergoing IVF. *International Journal of Andrology*.

[10] Richter C. A., Birnbaum L. S., Farabollini F. (2007). *In vivo* effects of bisphenol A in laboratory rodent studies. *Reproductive Toxicology*.

[11] Caserta D., Bordi G., Ciardo F. (2013). The influence of endocrine disruptors in a selected population of infertile women. *Gynecological Endocrinology*.

[12] Santangeli S., Maradonna F., Olivotto I., Piccinetti C. C., Gioacchini G., Carnevali O. (2017). Effects of BPA on female reproductive function: The involvement of epigenetic mechanism. *General and Comparative Endocrinology*.

[13] Ding Z.-M., Jiao X.-F., Wu D. (2017). Bisphenol AF negatively affects oocyte maturation of mouse in vitro through increasing oxidative stress and DNA damage. *Chemico-Biological Interactions*.

[14] Ehrlich S., Williams P. L., Missmer S. A. (2012). Urinary bisphenol A concentrations and early reproductive health outcomes among women undergoing IVF. *Human Reproduction*.

[15] Bloom M. S., Kim D., vom Saal F. S. (2011). Bisphenol A exposure reduces the estradiol response to gonadotropin stimulation during in vitro fertilization. *Fertility and Sterility*.

[16] Fujimoto V. Y., Kim D., vom Saal F. S., Lamb J. D., Taylor J. A., Bloom M. S. (2011). Serum unconjugated bisphenol A concentrations in women may adversely influence oocyte quality during in vitro fertilization. *Fertility and Sterility*.

[17] Zhao H.-Y., Bi Y.-F., Ma L.-Y. (2012). The effects of bisphenol A (BPA) exposure on fat mass and serum leptin concentrations have no impact on bone mineral densities in non-obese premenopausal women. *Clinical Biochemistry*.

[18] Kandaraki E., Chatzigeorgiou A., Livadas S. (2011). Endocrine disruptors and Polycystic Ovary Syndrome (PCOS): Elevated serum levels of bisphenol A in women with PCOS. *The Journal of Clinical Endocrinology & Metabolism*.

[19] Buck Louis G. M., Sundaram R., Sweeney A. M., Schisterman E. F., Maisog J., Kannan K. (2014). Urinary bisphenol A, phthalates, and couple fecundity: the longitudinal investigation of fertility and the environment (LIFE) study. *Fertility and Sterility*.

[20] Wang B., Zhou W., Zhu W. (2018). Associations of female exposure to bisphenol A with fecundability: evidence from a preconception cohort study. *Environment International*.

[21] Fernández M., Bianchi M., Lux-Santos V., Libertun C. (2009). Neonatal exposure to bisphenol A alters reproductive parameters and gonadotropin releasing hormone signaling in female rats. *Environmental Health Perspectives*.

[22] Lee S. G., Kim J. Y., Chung J.-Y. (2013). Bisphenol a exposure during adulthood causes augmentation of follicular atresia and luteal regression by decreasing 17*β*-estradiol synthesis via downregulation of aromatase in rat ovary. *Environmental Health Perspectives*.

[23] Monje L., Varayoud J., Muñoz-de-Toro M., Luque E. H., Ramos J. G. (2010). Exposure of neonatal female rats to bisphenol A disrupts hypothalamic LHRH pre-mRNA processing and estrogen receptor alpha expression in nuclei controlling estrous cyclicity. *Reproductive Toxicology*.

[24] Zhang T., Li L., Qin X.-S. (2014). Di-(2-ethylhexyl) phthalate and bisphenol A exposure impairs mouse primordial follicle assembly *in vitro*. *Environmental and Molecular Mutagenesis*.

[25] Miao M., Yuan W., Yang F. (2015). Associations between bisphenol a exposure and reproductive hormones among female workers. *International Journal of Environmental Research and Public Health*.

[26] Souter I., Smith K. W., Dimitriadis I. (2013). The association of bisphenol-A urinary concentrations with antral follicle counts and other measures of ovarian reserve in women undergoing infertility treatments. *Reproductive Toxicology*.

[27] Brannick K. E., Craig Z. R., Himes A. D. (2012). Prenatal exposure to low doses of bisphenol a increases pituitary proliferation and gonadotroph number in female mice offspring at birth. *Biology of Reproduction*.

[28] Rodríguez H. A., Santambrosio N., Santamaría C. G., Muñoz-de-Toro M., Luque E. H. (2010). Neonatal exposure to bisphenol A reduces the pool of primordial follicles in the rat ovary. *Reproductive Toxicology*.

[29] Jukic A. M., Calafat A. M., Robert McConnaughey D. (2016). Urinary concentrations of phthalate metabolites and bisphenol a and associations with follicular-phase length, luteal-phase length, fecundability, and early pregnancy loss. *Environmental Health Perspectives*.

[30] Fernández M., Bourguignon N., Lux-Lantos V., Libertun C. (2010). Neonatal exposure to bisphenol A and reproductive and endocrine alterations resembling the polycystic ovarian syndrome in adult rats. *Environmental Health Perspectives*.

[31] Diamanti-Kandarakis E., Bourguignon J. P., Giudice L. C. (2009). Endocrine-disrupting chemicals: an Endocrine Society scientific statement. *Endocrine Reviews*.

[32] Mahoney M., Padmanabhan V. (2010). Developmental programming: Impact of fetal exposure to endocrine-disrupting chemicals on gonadotropin-releasing hormone and estrogen receptor mRNA in sheep hypothalamus. *Toxicology and Applied Pharmacology*.

[33] Brown M. A., Chang R. J. (2007). Polycystic ovary syndrome: Clinical and imaging features. *Ultrasound Quarterly*.

[34] Akin L., Kendirci M., Narin F. (2015). The endocrine disruptor bisphenol A may play a role in the aetiopathogenesis of polycystic ovary syndrome in adolescent girls. *Acta Paediatrica*.

[35] Kandaraki E., Chatzigeorgiou A., Livadas S. (2011). Endocrine disruptors and Polycystic ovary syndrome (PCOS): Elevated serum levels of bisphenol A in women with PCOS. *The Journal of Clinical Endocrinology & Metabolism*.

[36] Newbold R. R., Jefferson W. N., Padilla-Banks E. (2009). Prenatal Exposure to Bisphenol A at environmentally relevant doses adversely affects the murine female reproductive tract later in life. *Environmental Health Perspectives*.

[37] Pan X., Wang X., Sun Y., Dou Z., Li Z. (2015). Inhibitory effects of preimplantation exposure to bisphenol-A on blastocyst development and implantation. *International Journal of Clinical and Experimental Medicine*.

[38] Newbold R. R., Jefferson W. N., Padilla-Banks E. (2007). Long-term adverse effects of neonatal exposure to bisphenol A on the murine female reproductive tract. *Reproductive Toxicology*.

[39] Signorile P. G., Spugnini E. P., Mita L. (2010). Pre-natal exposure of mice to bisphenol A elicits an endometriosis-like phenotype in female offspring. *General and Comparative Endocrinology*.

[40] Varayoud J., Ramos J. G., Bosquiazzo V. L., Lower M., Muñoz-de-Toro M., Luque E. H. (2011). Neonatal exposure to bisphenol A alters rat uterine implantation-associated gene expression and reduces the number of implantation sites. *Endocrinology*.

[41] Bromer J. G., Zhou Y., Taylor M. B., Doherty L., Taylor H. S. (2010). Bisphenol-A exposure in utero leads to epigenetic alterations in the developmental programming of uterine estrogen response. *The FASEB Journal*.

[42] Xiao S., Diao H., Smith M. A., Song X., Ye X. (2011). Preimplantation exposure to bisphenol A (BPA) affects embryo transport, preimplantation embryo development, and uterine receptivity in mice. *Reproductive Toxicology*.

[43] Buck Louis G. M., Peterson C. M., Chen Z. (2013). Bisphenol A and phthalates and endometriosis: the endometriosis: natural history, diagnosis and outcomes study. *Fertility and Sterility*.

[44] Borman E. D., Foster W. G., Greenacre M. K. E., Muir C. C., Decatanzaro D. (2015). Stress lowers the threshold dose at which bisphenol A disrupts blastocyst implantation, in conjunction with decreased uterine closure and e-cadherin. *Chemico-Biological Interactions*.

[45] Crawford B. R., deCatanzaro D. (2012). Disruption of blastocyst implantation by triclosan in mice: impacts of repeated and acute doses and combination with bisphenol-A. *Reproductive Toxicology*.

[46] Ehrlich S., Williams P. L., Missmer S. A. (2012). Urinary bisphenol A concentrations and implantation failure among women undergoing in vitro fertilization. *Environmental Health Perspectives*.

[47] Sugiura-Ogasawara M., Ozaki Y., Sonta S.-I., Makino T., Suzumori K. (2005). Exposure to bisphenol A is associated with recurrent miscarriage. *Human Reproduction*.

[48] Cantonwine D., Meeker J. D., Hu H. (2010). Bisphenol a exposure in Mexico City and risk of prematurity: a pilot nested case control study. *Environmental Health*.

[49] Moral R., Wang R., Russo I. H., Lamartiniere C. A., Pereira J., Russo J. (2008). Effect of prenatal exposure to the endocrine disruptor bisphenol A on mammary gland morphology and gene expression signature. *Journal of Endocrinology*.

[50] Caserta D., Ciardo F., Bordi G. (2013). Correlation of endocrine disrupting chemicals serum levels and white blood cells gene expression of nuclear receptors in a population of infertile women. *International Journal of Endocrinology*.

[51] Chao H.-H., Zhang X.-F., Chen B. (2012). Bisphenol A exposure modifies methylation of imprinted genes in mouse oocytes via the estrogen receptor signaling pathway. *Histochemistry and Cell Biology*.

[52] Xu J., Osuga Y., Yano T. (2002). Bisphenol A induces apoptosis and G2-to-M arrest of ovarian granulosa cells. *Biochemical and Biophysical Research Communications*.

[53] Miao M., Yuan W., Zhu G. (2011). In utero exposure to bisphenol-A and its effect on birth weight of offspring. *Reproductive Toxicology*.

[54] Chou W.-C., Chen J.-L., Lin C.-F., Chen Y.-C., Shih F.-C., Chuang C.-Y. (2011). Biomonitoring of bisphenol A concentrations in maternal and umbilical cord blood in regard to birth outcomes and adipokine expression: a birth cohort study in Taiwan. *Environmental Health*.

[55] Philippat C., Mortamais M., Chevrier C. (2012). Exposure to phthalates and phenols during pregnancy and offspring size at birth. *Environmental Health Perspectives*.

[56] Padmanabhan V., Siefert K., Ransom S. (2008). Maternal bisphenol-A levels at delivery: A looming problem?. *Journal of Perinatology*.

[57] Wolff M. S., Engel S. M., Berkowitz G. S. (2008). Prenatal phenol and phthalate exposures and birth outcomes. *Environmental Health Perspectives*.

[58] Lassen T. H., Frederiksen H., Jensen T. K. (2014). Urinary bisphenol a levels in young men: Association with reproductive hormones and semen quality. *Environmental Health Perspectives*.

[59] Hejmej A., Kotula-Balak M., Bilinsk B. (2011). Antiandrogenic and estrogenic compounds: effect on development and function of male reproductive system. *Steroids – Clinical Aspect*.

[60] Meeker J. D., Yang T., Ye X., Calafat A. M., Hauser R. (2011). Urinary concentrations of parabens and serum hormone levels, semen quality parameters, and sperm DNA damage. *Environmental Health Perspectives*.

[61] Akingbemi B. T., Sottas C. M., Koulova A. I., Klinefelter G. R., Hardy M. P. (2004). Inhibition of testicular steroidogenesis by the xenoestrogen bisphenol A is associated with reduced pituitary luteinizing hormone secretion and decreased steroidogenic enzyme gene expression in rat Leydig cells. *Endocrinology*.

[62] Knez J., Kranvogl R., Breznik B. P., Vončina E., Vlaisavljević V. (2014). Are urinary bisphenol A levels in men related to semen quality and embryo development after medically assisted reproduction?. *Fertility and Sterility*.

[63] Rahman M. S., Kwon W., Lee J., Yoon S., Ryu B., Pang M. (2015). Bisphenol-A affects male fertility via fertility-related proteins in spermatozoa. *Scientific Reports*.

[64] Hulak M., Gazo I., Shaliutina A., Linhartova P. (2013). In vitro effects of bisphenol A on the quality parameters, oxidative stress, DNA integrity and adenosine triphosphate content in sterlet (Acipenser ruthenus) spermatozoa. *Comparative Biochemistry and Physiology - C Toxicology and Pharmacology*.

[65] Tiwari D., Vanage G. (2013). Mutagenic effect of Bisphenol A on adult rat male germ cells and their fertility. *Reproductive Toxicology*.

[66] Vilela J., Hartmann A., Silva E. F. (2014). Sperm impairments in adult vesper mice (Calomys laucha) caused by in utero exposure to bisphenol A. *Andrologia*.

[67] Wang P., Luo C., Li Q., Chen S., Hu Y. (2014). Mitochondrion-mediated apoptosis is involved in reproductive damage caused by BPA in male rats. *Environmental Toxicology and Pharmacology*.

[68] O'Connell M., McClure N., Lewis S. E. M. (2002). The effects of cryopreservation on sperm morphology, motility and mitochondrial function. *Human Reproduction*.

[69] Chen M., Tang R., Fu G. (2013). Association of exposure to phenols and idiopathic male infertility. *Journal of Hazardous Materials*.

[70] Meeker J. D., Calafat A. M., Hauser R. (2010). Urinary bisphenol A concentrations in relation to serum thyroid and reproductive hormone levels in men from an infertility clinic. *Environmental Science & Technology*.

[71] Nakamura D., Yanagiba Y., Duan Z. (2010). Bisphenol A may cause testosterone reduction by adversely affecting both testis and pituitary systems similar to estradiol. *Toxicology Letters*.

[72] Mendiola J., Jørgensen N., Andersson A.-M. (2010). Are environmental levels of bisphenol A associated with reproductive function in fertile men?. *Environmental Health Perspectives*.

[73] Pasqualotto F. F., Sharma R. K., Nelson D. R., Thomas A. J., Agarwal A. (2000). Relationship between oxidative stress, semen characteristics, and clinical diagnosis in men undergoing infertility investigation. *Fertility and Sterility*.

[74] Nishikawa M., Iwano H., Yanagisawa R., Koike N., Inoue H., Yokota H. (2010). Placental transfer of conjugated bisphenol A and subsequent reactivation in the rat fetus. *Environmental Health Perspectives*.

[75] Lee Y. J., Ryu H.-Y., Kim H.-K. (2008). Maternal and fetal exposure to bisphenol A in Korea. *Reproductive Toxicology*.

[76] Bloom M. S., vom Saal F. S., Kim D., Taylor J. A., Lamb J. D., Fujimoto V. Y. (2011). Serum unconjugated bisphenol A concentrations in men may influence embryo quality indicators during in vitro fertilization. *Environmental Toxicology and Pharmacology*.

[77] Li D.-K., Zhou Z., Miao M. (2010). Relationship between urine bisphenol-A Level and declining male sexual function. *Journal of Andrology*.

[78] Li D., Zhou Z., Qing D. (2010). Occupational exposure to bisphenol-A (BPA) and the risk of self-reported male sexual dysfunction. *Human Reproduction*.

[79] Komarowska M. D., Milewski R., Charkiewicz R. (2016). Are anti-Müllerian hormone and its receptor polymorphism associated with the hormonal condition of undescended testes?. *Advances in Medical Sciences*.

[80] Matuszczak E., Hermanowicz A., Komarowska M. (2013). Serum AMH in physiology and pathology of male gonads. *International Journal of Endocrinology*.

[81] Komarowska M. D., Hermanowicz A., Matuszczak E. (2012). Anti-Müllerian hormone levels in serum 1 year after unilateral orchiopexy. *Journal of Pediatric Endocrinology and Metabolism*.

[82] Hermanowicz A., Matuszczak E., Debek W. (2012). Expression of estrogen receptors *α* and *β* in paratesticular tissues in boys operated on for unilateral cryptorchidism between the 1st and 4th years of life. *Medical Science Monitor*.

[83] Virtanen H. E., Koskenniemi J. J., Sundqvist E. (2012). Associations between congenital cryptorchidism in newborn boys and levels of dioxins and PCBs in placenta. *International Journal of Andrology*.

[84] Hosie S., Loff S., Witt K., Niessen K., Waag K.-L. (2000). Is there a correlation between organochlorine compounds and undescended testes?. *European Journal of Pediatric Surgery*.

[85] Fénichel P., Déchaux H., Harthe C. (2012). Unconjugated bisphenol A cord blood levels in boys with descended or undescended testes. *Human Reproduction*.

[86] Chung E., Brock G. B. (2013). Cryptorchidism and its impact on male fertility: a state of art review of current literature. *Canadian Tax Journal*.

[87] Wilkerson M. L., Bartone F. F., Fox L. (2001). Fertility potential: a comparison of intra-abdominal and intracanalicular testes by age groups in children. *Hormone Research*.

[88] Tasian G. E., Hittelman A. B., Kim G. E., DiSandro M. J., Baskin L. S. (2009). Age at orchidopexy and testis palpability predict germ cell and leydig cell loss: clinical predictors of adverse histological features of cryptorchidism. *The Journal of Urology*.

[89] Dodge L. E., Williams P. L., Williams M. A. (2015). Paternal urinary concentrations of parabens and other phenols in relation to reproductive outcomes among couples from a fertility clinic. *Environmental Health Perspectives*.

[90] Radwan M., Wielgomas B., Dziewirska E. (2018). Urinary bisphenol A levels and male fertility. *American Journal of Men's Health*.

[91] Tomza-Marciniak A., Stępkowska P., Kuba J., Pilarczyk B. (2018). Effect of bisphenol A on reproductive processes: a review of in vitro, in vivo and epidemiological studies. *Journal of Applied Toxicology*.

